# Editorial: The fundamental biology of basophils in health and disease

**DOI:** 10.3389/fimmu.2023.1292279

**Published:** 2023-10-18

**Authors:** Christophe Pellefigues, Hajime Karasuyama

**Affiliations:** ^1^ Université Paris Cité, Centre de Recherche sur l’Inflammation, Institut National de la santé et de la recherche médicale (INSERM) UMR1149, Centre national de la recherche scientifique (CNRS) EMR8252, Faculté de Médecine site Bichat, Paris, France; ^2^ Université Paris Cité, Laboratoire d’Excellence Inflamex, Paris, France; ^3^ Inflammation, Infection and Immunity Laboratory, TMDU Advanced Research Institute, Tokyo Medical and Dental University (TMDU), Tokyo, Japan

**Keywords:** basophil, health, disease, IgE, allergy, MRGPRX2, urticaria, dermatitis

## 1 Quick history of basophils research and emerging hot topics

**Figure 1:**
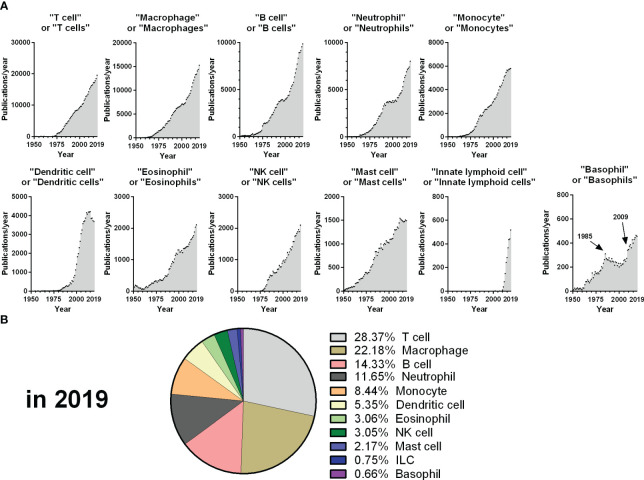
Evolution of publications from 1950 to 2019 for each main immune cell type. **(A)** The number of publications per year retrieved from the Pubmed database from 1950 to 2019 is depicted for each query, corresponding to the dot plot titles. **(B)** Percentage of publications retrieved from each query from the sum of all the queries represented in A for the year 2019. The year 2019 was chosen as an endpoint as the volume and dynamics of publications changed drastically from 2020 due to the COVID-19 pandemic.

Basophils are one of the rarest immune cell types, representing less than 1% of circulating leucocytes in humans. They were discovered more than 140 years ago by Paul Ehrlich, but basophils research has suffered from their rarity and from “shifting trends” in immunology. Indeed, from 1985 to 2009, the number of publications on basophils stalled (**Figure 1A**), while “newer” immune cells grabbed a steady focus (i.e., “Dendritic cells”). The year 2009 saw a renewal of basophils research, which may have arisen from several anterior breakthroughs, beginning with thorough descriptions of the regulation and dynamics of basophil degranulation (1–6), of their expression of IL-4 (7, 8), and of human basophils promoting B cell IgE production without exogenous IL-4 (9) in the 1980s-1990s. The democratization of flow cytometry in the 2000s enabled better protocols of purification and deeper characterization of the human basophil (10, 11), which fostered the development of the Basophil Activation Test (BAT) (12). Mice basophil research showed that basophils are a primary source of IL-4 in helminth infection (13), mediate delayed hypersensitivity reactions after intravenous IgE sensitization and intradermal allergen challenge (14), and promote *in vivo* antibody responses (15), Th2 responses (16), and IgG-driven anaphylaxis (17). This formed the announcement of a “rebirth” of basophils research in 2009, with numerous major publications characterizing how basophils are activated and promote Th2 responses (18–27).

From 2009 to 2019, the number of publications citing basophils rose steadily, with many discoveries deciphering the fundamental biology of basophils and their contribution to health or disease. This was supported by the generation of specific basophil-deficient mice (28, 29) and conditionally basophil-deficient mice (30, 31), which allowed unambiguous demonstrations of the various roles of basophils (32). Nowadays, we have a better understanding of several aspects of basophils biology, including their differentiation (and a pre-mature basophil state) (33–35), their heterogeneity (36–38), their responsiveness to various ligands (24, 27, 37–39), their expression of chemokine receptors (40, 41), and the mechanisms by which they can present antigens (42, 43). The controversies regarding how basophils promote the priming of T cells are underlined by Möbs et al.

A deleterious role of basophils seems evident in several allergic diseases of the skin (atopic dermatitis and chronic spontaneous urticaria), the airways (asthma and chronic rhinosinusitis), or the gut and in some anaphylactic reactions. Basophils are also detrimental in various autoimmune diseases (i.e., systemic lupus erythematosus) and chronic inflammatory or fibrotic diseases of the lungs (chronic obstructive pulmonary dysfunction), the gut (inflammatory bowel diseases), the kidneys (ischemia/reperfusion-induced fibrosis), or the heart (allograft-induced fibrosis) (32). Wiebe et al. underlined how basophils contribute to pruritus in allergic and inflammatory or autoimmune skin diseases. An updated description of the contribution of basophils to non-allergic and non-parasitic diseases, with a focus on autoimmune and chronic inflammatory disorders, has been reviewed by Poto et al. Basophils show complex capabilities to promote tumor progression or, inversely, tumor suppression. An updated description of the potential prognostic value of circulating basophils counts and a summary of their functions in various cancer or cancer models has been presented in another manuscript by Poto et al.

These complex roles in cancer highlight that basophils can also promote health and homeostasis in a broad array of conditions: they display unique interactions with hematopoietic and non-hematopoietic cells during lung development (38); they secrete both retinoic acid (44), IL-10 (45), and cleave extracellular ATP (46) to reduce inflammation; and they promote the resolution of infectious and sterile inflammation in the skin, liver, lungs, or heart (32, 47). Basophils have also emerged as being protective in infectious models beyond ectoparasite infections, including in a mouse model of sepsis (48) and of malarial infection (49, 50).

## 2 Original research, brief reports, and hypotheses

Despite these exciting discoveries, basophils remain the least studied of the main immune cells, representing less than 1% of these publications in 2019 (**Figure 1B**). In this context, the aim of this Research Topic was to aggregate original manuscripts exploring emerging hot topics in basophils research, which will be presented below.

IgE crosslinking induces several signaling events controlling intracellular calcium mobilization and degranulation. Hansen Selnø et al. explored the expression of the sarcoplasmic reticulum Ca2+ ATPase (SERCA2) in human basophils and its function. SERCA2 expression is strongly inversely correlated with anti-IgE-induced histamine release, and pharmaceutical inhibition or activation of SERCA proteins controls the amplitude of basophil histamine release. Thus, SERCA2 appears as a new negative regulator of basophil degranulation.

Basophil responsiveness to IgE decreases among patients suffering from chronic spontaneous urticaria (CSU), supposedly due to the presence of autoreactive antibodies against IgE or its receptor. However, Matsubara et al. showed that the response of CSU patients’ basophils to the anaphylatoxin C5a is unaltered. This suggests targeting the C5a/C5aR axis may be of critical value in patients refractory to anti-FcϵRIα treatments.

In chronic inducible urticaria, urticaria is induced by specific stimuli, such as ultraviolet light exposure. Mizuno et al. showed that the circulating basophils of these patients are more activated than those of healthy controls (as CSU patients) but without any IgE hyporesponsiveness. This highlights differences in the pathophysiology of these distinct conditions.

Peripheral basopenia in CSU patients is associated with disease activity, and basophils are found in patients’ skin lesions. Kishimoto et al. confirmed previous reports underlining a reversal of basopenia upon treatment of CSU patients with Omalizumab or anti-histamine. Then, using an oxazolone-induced contact dermatitis model, they demonstrated that the migration of circulating basophils to skin lesions provoked a transient basopenia. This supports the concept that clinical observations of basopenia reflect an active basophils extravasation.

El Hachem et al. explored the mechanisms governing basophils extravasation in the skin in FITC-induced dermatitis. They revealed that basophil migration was critically dependent on the secretion of IL-3 by T cells. They also demonstrated that IL-3-stimulated human and mice basophils relied on an autocrine retinoid acid production to drive their expression of specific integrins and mice basophil extravasation. Overall, these results strongly suggest that T cell IL-3 drives basophils autocrine secretion of retinoic acid to enable their extravasation in the inflamed skin.

The unique properties of skin-homing basophils have been described in a hypothesis and theory article by Shibuya and Kim, which suggests these basophils may have a unique identity, acquired during hematopoiesis and/or through late imprinting by the action of TSLP and epithelial-derived alarmins as mice lung basophils do under the control of IL-33 and GM-CSF (38). Skin-infiltrating basophils may externalize MRGPRX2, a receptor involved in pseudoallergic reactions and neuroimmune interactions.

MRGPRX2 expression by basophils has been the subject of some controversy. In this Research Topic, Toscano et al. explored the expression and function of this receptor on basophils from patients allergic to birch pollen or hypersensitive to moxifloxacin. Circulating basophils express only very low levels of functional surface MRGPRX2, but this is very quickly externalized by specific activation (anti-IgE, fMLP) or non-specific activation (i.e., purification). Thus, the reactivity of patients’ basophils to MRGPRX2 ligands can be studied but only when using carefully controlled conditions.

Basophils are known to participate in allergic airway inflammation and allergic asthma but have mainly been studied following allergen challenge or asthma exacerbation. Here, Iype et al. analyzed the expression of activation markers on stable asthmatics basophils and reported that they express more surface CD25 but no other activation markers. As human basophils activated by IL-2 secrete type 2 cytokines, and IL-2 is associated with asthma, this pathway seems important in asthma chronicity and pathophysiology.

The development of efficient helminth vaccines is an ongoing challenge in immunology. Thuma et al. cloned a new immunogenic protein secreted by the model helminth *Nippostrongylus brasiliensis* (*Nb*) during infection, Nb-LSA1a. Immunization with Nb-LSA1a induces specific IgG1 and protective immunity against *Nb* infection in wild-type but not basophil-deficient mice. This strongly suggests helminth vaccination strategies should benefit from inducing basophil-dependent immunity.

Overall, the manuscripts submitted to this Research Topic underline current and emerging trends in basophils immunology: the regulation of their degranulation via FcϵRIα or MRGPRX2; their roles in chronic urticaria, pruritic diseases, asthma, or cancer; the controversies surrounding their regulation of T cell polarization and their potency in promoting anti-helminth protective immunity; the mechanisms controlling their extravasation and peripheral basopenia; and, finally, the concept of mature basophils harboring distinct specific identities.

## Author contributions

CP: Writing – original draft, Writing – review & editing. HK: Writing – review & editing.
